# AmyR Is a Novel Negative Regulator of Amylovoran Production in *Erwinia amylovora*

**DOI:** 10.1371/journal.pone.0045038

**Published:** 2012-09-18

**Authors:** Dongping Wang, Schuyler S. Korban, P. Lawrence Pusey, Youfu Zhao

**Affiliations:** 1 Department of Crop Sciences, University of Illinois at Urbana-Champaign, Urbana, Illinois, United States of America; 2 Department of Natural Resources and Environmental Sciences, University of Illinois at Urbana-Champaign, Urbana, Illinois, United States of America; 3 Tree Fruit Research Laboratory, USDA-Agriculture Research Service, Wenatchee, Washington, United States of America; University of Wisconsin-Milwaukee, United States of America

## Abstract

In this study, we attempted to understand the role of an orphan gene *amyR* in *Erwinia amylovora*, a functionally conserved ortholog of *ybjN* in *Escherichia coli*, which has recently been characterized. Amylovoran, a high molecular weight acidic heteropolymer exopolysaccharide, is a virulent factor of *E. amylovora*. As reported earlier, amylovoran production in an *amyR* knockout mutant was about eight-fold higher than that in the wild type (WT) strain of *E. amylovora*. When a multicopy plasmid containing the *amyR* gene was introduced into the *amyR* mutant or WT strains, amylovoran production was strongly inhibited. Furthermore, amylovoran production was also suppressed in various amylovoran-over-producing mutants, such as *grrSA* containing multicopies of the *amyR* gene. Consistent with amylovoran production, an inverse correlation was observed between *in vitro* expression of *amyR* and that of amylovoran biosynthetic genes. However, both the *amyR* knockout mutant and over-expression strains showed reduced levan production, another exopolysaccharide produced by *E. amylovora*. Virulence assays demonstrated that while the *amyR* mutant was capable of inducing slightly greater disease severity than that of the WT strain, strains over-expressing the *amyR* gene did not incite disease on apple shoots or leaves, and only caused reduced disease on immature pear fruits. Microarray studies revealed that amylovoran biosynthesis and related membrane protein-encoding genes were highly expressed in the *amyR* mutant, but down-regulated in the *amyR* over-expression strains *in vitro*. Down-regulation of amylovoran biosynthesis genes in the *amyR* over-expression strain partially explained why over-expression of *amyR* led to non-pathogenic or reduced virulence *in vivo*. These results suggest that AmyR plays an important role in regulating exopolysaccharide production, and thus virulence in *E. amylovora*.

## Introduction

Fire blight, caused by the bacterium *Erwinia amylovora*, is the most devastating bacterial disease on apples and pears, which results in annual economic losses of around $100 million in the United States alone [1]. As a member of the family *Enterobacteriacae*, *E. amylovora* is closely related to many important human and animal pathogens such as *Escherichia coli*, *Salmonella enterica*, *Shigella flexineri* and *Yersinia pestis*. Like many other Gram-negative pathogenic bacteria, *E. amylovora* utilizes a type III secretion system (T3SS) to cause disease [2], [3], [4]. The hypersensitive response (HR) and pathogenicity (*hrp*)-T3SS gene cluster is essential for *E. amylovora* to elicit an HR in non-host plants and cause disease in host plants. Most genes in the *hrp* cluster are controlled by HrpL, a member of the ECF subfamily of sigma factors [5]. In turn, the expression of *hrpL* is activated by the HrpS sigma 54 enhancer-binding protein. HrpL recognizes promoters (*hrp* boxes) of genes such as *hrpA*, encoding a major component of the needle structure, as well as effector genes such as *avrRpt2*, *dspE*, *hrpW*, and *hrpN* [6], [7].

*E. amylovora* produces two types of exopolysaccharides (EPS), amylovoran and levan, as virulence factors [8], [9]. Amylovoran is a high molecular weight acidic heteropolymer, composed of a pentasaccharide repeating unit containing four galactose residues and one glucuronic acid molecule [10]; whereas, levan is a simple homopolymer of fructose residues. While mutants deficient in amylovoran biosynthesis are nonpathogenic [11], [12], mutants deficient in levan production are reduced in virulence [13]. In *E. amylovora*, amylovoran biosynthetic genes are encoded by the *ams* operon, consisting of 12 genes, from *amsA* to *amsL*, with *amsG* as the first gene in the operon [11], while levan is synthesized by levansucrase encoded by the gene *lsc* [9], [14].

In a previous study, we have identified two-component signal transduction (TCST) mutants exhibiting varying levels of amylovoran production *in vitro* [8]. Among them, the most important one is the Rcs phosphorelay system, which acts as a positive regulator of amylovoran production and is required for pathogenicity [8], [12], [15], [16]. Furthermore, amylovoran production is also negatively regulated by other TCSTs, including EnvZ/OmpR and GrrA/GrrS systems [8]. These results suggest that TCSTs may form a complicated regulatory network to govern production of amylovoran. On the other hand, three activators, *rlsA*, *rlsB* and *rlsC*, have been reported for *lsc* expression [14], [17].

Previously, we have also reported that a mutation of *ybjN* gene in *E. amylovora*, an ortholog of *E. coli ybjN* gene, results in about eight-fold more amylovoran than that of the wild type strain, suggesting that this gene may act as a negative regulator of amylovoran production [8]. Thus, we have renamed this gene as *Amy*lovoran *R*epressor, *amyR* (*Eam_1300*). As one of the enterobacteria-specific orphan genes, *ybjN/amyR* has been initially annotated as either encoding a putative sensory transduction regulator protein or a predicted oxidoreductase [18], [19], [20].

Characterization of the *E. coli ybjN* mutant revealed that *ybjN* mutation resulted in pleiotropic phenotypes, including increased motility, fimbriation (auto-aggregation), exopolysaccharide production, and biofilm formation in *E. coli*. In contrast, over-expression of *ybjN* (in terms of multiple copies) resulted in reduced motility, fimbriation, exopolysaccharide production, biofilm formation and acid resistance [20]. Our findings also indicated that *amyR* from *E. amylovora* is functionally conserved with *E. coli ybjN*, suggesting similar evolution of the YbjN family proteins in enterobacteria. Phenotypic data along with transcriptomic profiling suggest that YbjN in *E. coli* may play important roles in regulating bacterial multicellular behavior, metabolism, and survival under stress conditions [20].

The overall goal of this study was to determine the regulatory role(s) of AmyR in the pathogenicity of *E. amylovora*. Phenotypic characterization of a mutant as well as over-expression of *amyR* in wild-type strain of *E. amylovora* indicated that *amyR* acts as a general suppressor of virulence factors, and thus of virulence in host plants. Transcriptomic profiling has determined the regulon of AmyR in *E. amylovora*.

## Results

### AmyR negatively regulates amylovoran production

**Figure 1:**
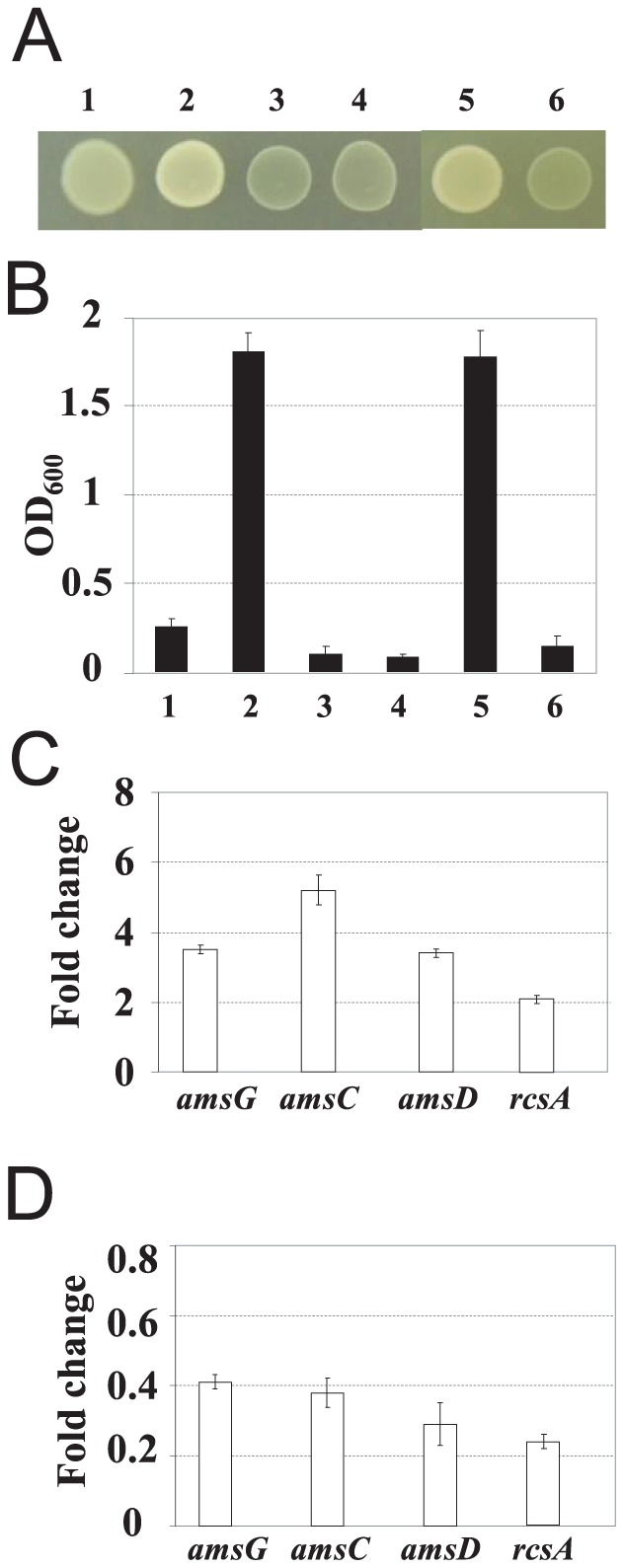
AmyR regulates amylovoran production and gene expression. A. Growth of *Erwinia amylovora* wild type (WT), *amyR* mutant, complementation strain and WT containing pAmyR2 plasmid on Luria-Bertani plates. Pictures were taken at 24 h post-inoculation. pAmyR2: plasmid containing 1.1-kb PCR fragment of *Erwinia amyR* gene and promoter in pGEM T-easy vector. **B**. Amylovoran production of *E. amylovora* wild type (WT), *amyR* mutant, complementation strain and WT containing pAmyR2 plasmid *in vitro*. Bacterial strains were grown in MBMA media with 1% sorbitol for 24 h at 28°C with shaking. The amount of amylovoran was measured with the CPC assay and normalized to a cell density of 1. Data points represent means of three replicates ± standard deviations. Similar results were obtained in three independent experiments. 1: Ea1189, 2: Δ*amyR*, 3: Δ*amyR* (pAmyR2), 4: Δ*amyR* (pAmyR3), 5: Ea273, 6: Ea273 (pAmyR2). **C**. Relative quantification of amylovoran biosynthetic genes in *amyR* mutant compared to WT strain by qRT-PCR. Bacterial cells were grown in MBMA with 1% sorbitol for 18 hours at 28°C with shaking. **D**. Relative quantification of amylovoran biosynthetic genes in WT containing pAmyR2 plasmid compared to WT strain by qRT-PCR. Bacterial cells were grown in MBMA with 1% sorbitol for 18 hours at 28°C with shaking.

In a previous study, mutation of an *E. amylovora amyR* gene resulted in over-production of amylovoran by about eight-fold than that of the wild-type strain Ea1189 [8]. When grown on LB plate, the mutant exhibited a mucoid phenotype, indicating that the mutant produced more exopolysaccharide than that of EA1189 and of complementation strains with either high- or low-copy numbers of the *amyR* gene. Both Ea1189 and complementation strains were non-mucoid (Fig. 1A). Another amylovoran over-producing WT strain, Ea273, has been reported to be mucoid on LB plate [21]. When a high copy plasmid containing the *amyR* gene (pAmyR2) was introduced into Ea273, the strain became non-mucoid (Fig. 1A), suggesting that multicopies of the *amyR* gene suppressed amylovoran production.

Mucoid phenotypes were confirmed by quantitatively measuring amylovoran production in liquid MBMA medium as described previously [8], [12]. The *amyR* mutant had about 7 to 8-fold increase in amylovoran production compared to that of Ea1189 (Fig. 1B), and the amount produced by the mutant was similar to that of Ea273 [8]. Levels of amylovoran produced by both complementation strains and Ea273 harboring pAmyR2 were almost half of that produced by Ea1189 (Fig. 1B). These results further suggested that AmyR suppressed amylovoran production.

Expression of selected amylovoran biosynthesis and regulatory genes was further determined by quantitative RT-PCR in MBMA medium. Expression of *amsG*, *amsC* and *amsD* was three to five-fold higher in the *amyR* mutant than in Ea1189; while, expression of *amsG*, *amsC* and *amsD* in Ea1189 containing pAmyR2 was about two- to three- fold lower than that of Ea1189 (Fig. 1CD). Expression of the *rcsA* gene, a rate-limiting factor in amylovoran production [22], in the *amyR* mutant increased by two-fold in the *amyR* mutant compared to Ea1189; while, expression of *rcsA* decreased by 4.5 fold in Ea1189 containing pAmyR2 as compared to that of Ea1189 (Fig. 1CD). As expected, no expression of the *amyR* gene in the mutant was detected. In contrast, expression of the *amyR* gene in Ea1189 and Ea273 containing pAmyR2 increased 13.4 and 22.3 fold as compared to those in Ea1189 and in Ea273 strains, respectively. These results demonstrated that AmyR was a negative regulator of amylovoran production by regulating *ams* gene expression. These findings also suggested that AmyR might negatively regulate amylovoran production by influencing *rcsA* gene expression.

### Multicopies of the *amyR* gene suppress amylovoran production in various mutant strains

**Figure 2:**
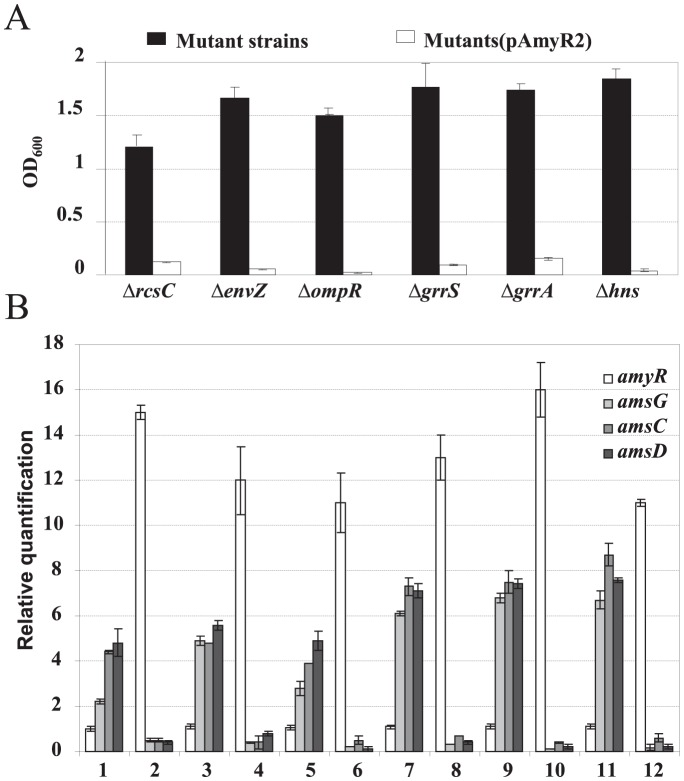
Over-expression of the *amyR* gene suppresses amylovoran production in various amylovoran over-producing mutants. **A**. Amylovoran production of various mutant strains with or without pAmyR2 plasmid. Bacterial strains were grown in MBMA media with 1% sorbitol for 24 hours at 28°C with shaking. The amount of amylovoran was measured with the CPC assay and normalized to a cell density of 1. **B**. Relative quantification of *amyR* and amylovoran biosynthetic genes in various mutant strains with or without pAmyR2 plasmid by qRT-PCR. Bacterial strains were grown in MBMA media with 1% sorbitol for 18 hours at 28°C with shaking. 1: Δ*rcsC*, 2: Δ*rcsC* (pAmyR2), 3: Δ*envZ*, 4: Δ*envZ* (pAmyR2), 5: Δ*ompR*, 6: Δ*ompR* (pAmyR2), 7: Δ*grrS*, 8: Δ*grrS* (pAmyR2), 9: Δ*grrA*, 10: Δ*grrA* (pAmyR2), 11: Δ*hns*, 12: Δ*hns* (pAmyR2).

Previously, we have reported that many mutants in Ea1189 background, including *rcsC*, *envZ*, *ompR*, *grrS*, *grrA* and *hns*, produce 6- to 10-fold higher levels of amylovoran than that of Ea1189 (Fig. 2A) [8]. When the *amyR* gene in a multi-copy plasmid was introduced into these mutant strains, transformants harboring pAmyR2 produced either similar amounts of amylovoran to those of Ea1189 or did not produce any amylovoran (Fig. 2A). These results suggested that AmyR may act downstream of these regulatory genes in regulating amylovoran production.

Quantitative RT-PCR was also conducted to determine the relative expression of *amyR*, *amsG*, *amsC* and *amsD* genes in Ea1189, mutant strains, as well as mutants containing pAmyR2. Consistent with amylovoran production, *amsG*, *amsC* and *amsD* genes were significantly up-regulated by 2- to 9-fold in these mutant strains (Fig. 2B). However, levels of expression of the *amyR* gene itself in *rcsC*, *envZ*, *ompR*, *grrS*, *grrA* and *hns* mutants were similar to those of the Ea1189 strain (Fig. 2B). This suggested that up-regulation of *ams* gene expression was not due to the *amyR* gene in these mutants. Expression of the *amyR* gene in mutants containing pAmyR2 increased by 11- to16-fold compared to those of Ea1189; whereas, expression of *amsG*, *amsC* and *amsD* decreased by 5- to100- fold in mutants containing pAmyR2 as compared to Ea1189 (Fig. 2B). These results suggested that AmyR might function independently of these signaling pathways.

### AmyR affects virulence in *E. amylovora*

**Figure 3:**
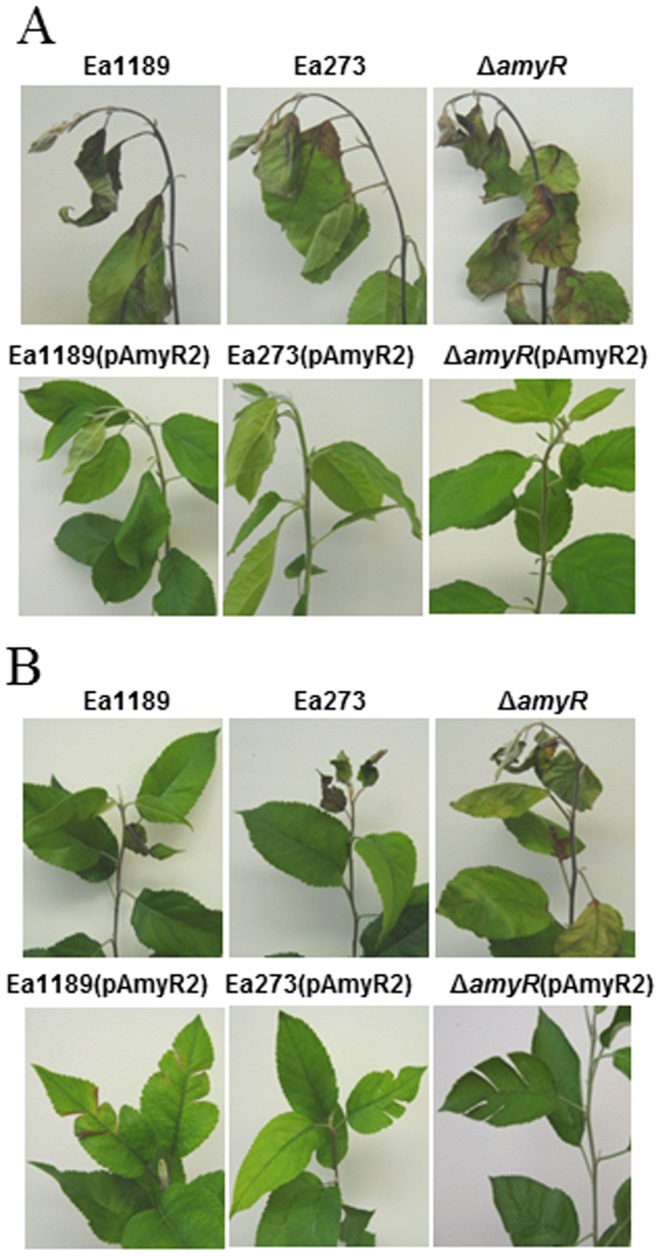
Virulence assays on apple shoots. **A**. Symptoms caused by *Erwinia amylovora* wild type (WT), *amyR* mutant, complementation strain and WT containing pAmyR2 plasmid on shoots of apple cv. Gala at 8 dpi. Virulence assays were performed using young annual shoots by pricking the tip with a needle, and pipetting 5 µl of pathogen suspension (OD_600_ = 0.1) onto the wounded tissue. The experiment was performed at least three times with similar results. **B**. Symptoms caused by WT and various strains on leaves of apple cv. Red Delicious at 8 dpi. Young leaves of Red Delicious plants were inoculated with scissors dipped in a bacterial suspension (OD_600_ = 0.1). dpi: days post inoculation.

**Figure 4:**
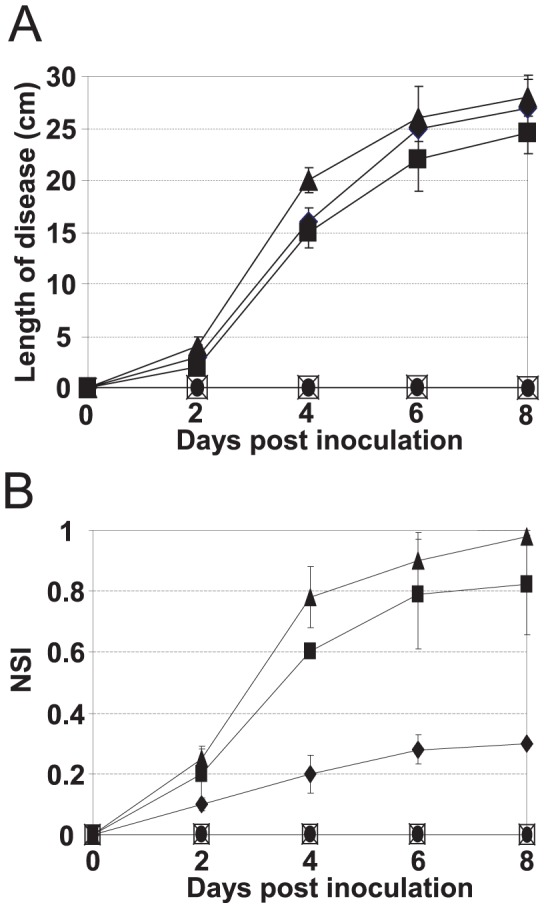
Disease severity of *Erwinia amylovora* wild-type (WT) strain, *amyR* mutant, complementation strains and WT containing pAmyR2 plasmid on apple plants. **A**. Gala shoots. **B**. Red Delicious leaves. NSI is the necrosis severity index calculated as described (Wang et al., 2010). ♦: Ea1189, ▪:Ea273, ▴: Δ*amyR*, •: Ea1189 (pAmyR2), □: Ea273 (pAmyR2), ×: Δ*amyR* (pAmyR2). Data points for Ea1189 (pAmyR2), Ea273 (pAmyR2) and *ΔamyR* (pAmyR2) overlap because no disease symptoms were observed.

As AmyR regulated amylovoran production, we then determined the role of AmyR in virulence. Virulence assay on Gala apple shoot was conducted as described previously [16]. Both Ea1189 and Ea273 caused visible necrosis around the inoculation site two days post-inoculation (dpi). The blackened symptoms rapidly moved along shoots, reaching 24.9 and 25.4 cm at 8 dpi for Ea1189 and Ea273, respectively (Fig. 3A and 4A). A visible rapid progress of disease symptom development was observed for the *amyR* mutant as compared to WT strains, particularly at 4 dpi where the average length of diseased shoots was 20.1, 15.0 and 15.4 cm for the *amyR* mutant, Ea1189, and Ea273, respectively (Fig. 4A). However, complementation and WT strains containing pAmyR2 were not capable of causing any disease symptoms on most inoculated apple shoots, but with a few exceptions (Fig. 3A); whereas complementation and WT strains containing pAmyR3 were greatly impaired in their abilities to cause disease. pAmyR3 is a plasmid containing *amyR* gene and promoter in a low copy vector. Disease progress was much slower and diseased tissue only reached 5.9 and 5.4 cm at 8 dpi for Ea1189 (pAmyR3) and Ea273 (pAmyR3), respectively (data not shown).

A virulence assay was also performed on leaves of a fire blight tolerant apple cv. Red Delicious [21]. As expected, Ea1189 caused necrosis on the inoculation site around 3 dpi, reaching the midrib at 4 dpi, and into the petiole at 8 dpi (Fig. 3B). The average disease index for Ea1189 was approximately 30% at 8 dpi (Fig. 4B). Ea273 strain caused greater disease severity than Ea1189 on Red Delicious as reported previously [21], with necrosis observed at 2 dpi and rapidly progressing into two adjacent leaves at 8 dpi. Deletion of *amyR* caused an increase in disease development with seven infected leaves observed at 8 dpi (Fig. 3B). Disease severity index for Ea273 and the *amyR* mutant was 81% and 98%, respectively (Fig. 4B). Again, no symptoms were observed for complementation and WT strains containing multicopies of *amyR* gene on leaves of Red Delicious (Figs. 3B, 4B).

**Figure 5:**
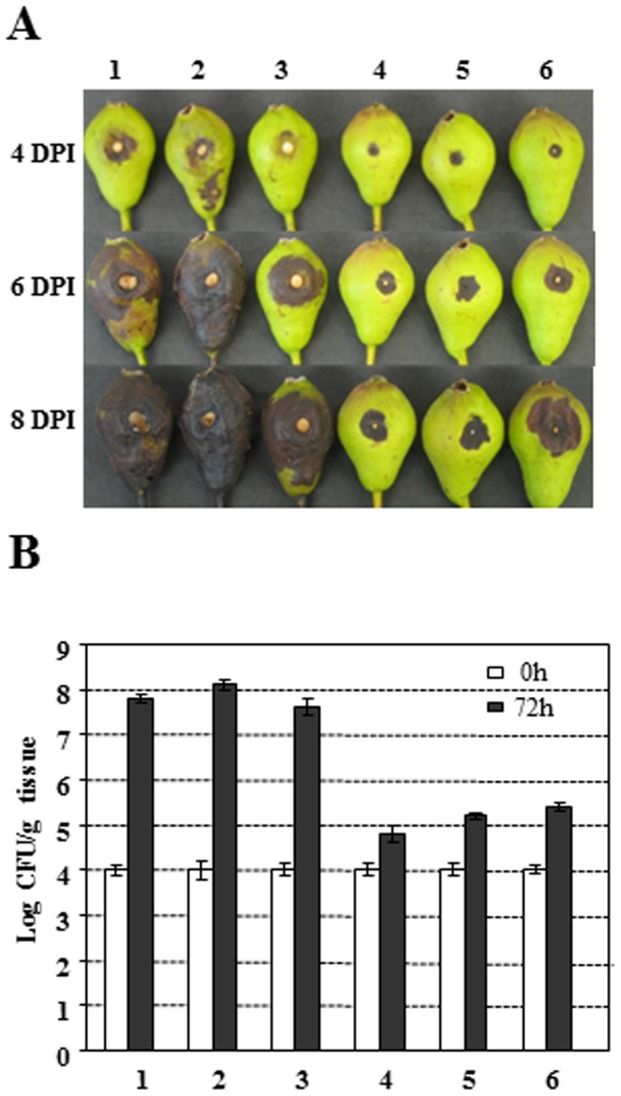
Virulence tests on immature pear fruits. **A**. Symptoms caused by *Erwinia amylovora* wild type (WT), *amyR* mutant, complementation strain and WT containing pAmyR2 plasmid on immature pear fruits. **B**. Bacterial growth on immature pear fruits. 1: Ea1189, 2: Δ*amyR*, 3: Δ*amyR* (pAmyR3), 4: Δ*amyR* (pAmyR2), 5: Ea1189 (pAmyR2), 6: Ea1189 (pAmyR3). Growth of bacterial strains was monitored at 0 and 72 h after inoculation. Data points represent means of three replicates ± standard errors. Similar results were obtained in repeated independent experiments.

In addition, virulence was tested on immature pear fruits. At 4 dpi, Ea1189 produced necrotic symptoms with visible bacterial ooze (Fig. 5A). At 6 dpi, increased necrotic lesions were observed and by 8 dpi, necrotic areas covered almost whole pear fruits. When immature pear fruits were inoculated with *amyR* mutants, disease symptoms were more severe than those observed for Ea1189 strain at 4 dpi, and necrotic symptoms progressed rapidly, covering whole pear fruit by 6 dpi. On the other hand, complementation of *amyR* mutant partially reduced its virulence to the level of WT. Moreover, disease progress was same to those caused by Ea1189 containing either pAmyR2 or pAmyR3 (Fig. 5A). When quantification of bacterial growth was determined at 3dpi, same levels of bacterial populations were detected for Ea1189, *amyR* mutant, and *amyR* (pAmyR3), but about 1000-fold greater than those for *amyR* (pAmyR2) and Ea1189 (pAmyR2 or pAmyR3) (Fig. 5B).

### Impact of the *amyR* gene on levan production

**Figure 6:**
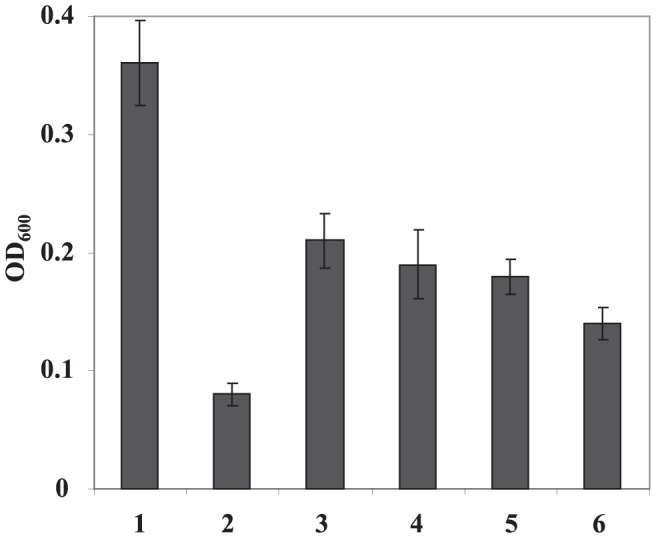
Levan production in *Erwinia amylovora* wild type (WT), *amyR* mutant, complementation strain and WT containing pAmyR2. All strains were grown overnight in LB broth at 28°C; 1 ml bacterial supernatant was mixed with equal volume of LS-buffer and incubated at 28°C. After 24 h, the amount of levan was quantified at OD_600_ and normalized to a cell density of 1. 1: Ea1189, 2: *ΔamyR*, 3: *ΔamyR* (pAmyR3), 4: ΔamyR (pAmyR2), 5: Ea1189 (pAmyR3), 6: Ea1189 (pAmyR2).

Levan production in the *amyR* mutant was around 4-fold lower than that of Ea1189 (Fig. 6). Complementation of the *amyR* mutant partially restored levan production. Whereas levan production in Ea1189 containing either pAmyR2 or pAmyR3 was about half of that detected in Ea1189. These results suggested that AmyR may negatively affect levan production in *E. amylovora*.

### Genes differentially expressed in *amyR* mutant and Ea1189 (pAmyR2) strain *in vitro*

To identify the AmyR regulon, microarray analyses were conducted to compare gene expression in the *amyR* mutant or Ea1189 (pAmyR2) with those of Ea1189 grown in MBMA. Under the treatment condition, no expression of the *amyR* gene was detected in the mutant; whereas, expression of the *amyR* gene was up-regulated by 15-fold in Ea1189 (pAmyR2) than in Ea1189 (Table 1), indicating the validity of mutant and *amyR* over-expression strains.

**Table 1:** Expression ratio of *amyR*, *ams*, *gal*, *rcs* and *lsc* genes in *amyR* mutant and over-expression strains compared to WT strain.

	Comparison		*ΔamyR*/WT	WT(pAmyR2)/WT
Gene	Name	Protein Description	MBMA	
*Eam_1300*	*amyR*	Putative sensory transduction regulator	0.01	15.4
*Eam_2171*	*amsA*	amylovoran biosynthesis tyrosine-protein kinase	2.42	0.48
*Eam_2170*	*amsB*	amylovoran biosynthesis glycosyltransferase	2.23	0.75
*Eam_2169*	*amsC*	amylovoran oligosaccharide repeat unit polymerase	4.24	0.55
*Eam_2168*	*amsD*	amylovoran biosynthesis glycosyltransferase	2.77	0.43
*Eam_2167*	*amsE*	amylovoran biosynthesis glycosyltransferase	2.84	0.69
*Eam_2166*	*amsF*	amylovoran biosynthesis protein	1.76	0.49
*Eam_2174*	*amsG*	UDP-galactose-lipid carrier transferase	2.80	0.51
*Eam_2173*	*amsH*	amylovoran export protein	3.00	0.68
*Eam_2172*	*amsI*	amylovoran biosynthesis protein-tyrosine-phosphatase	3.35	0.70
*Eam_2165*	*amsJ*	amylovoran biosynthesis protein	2.40	**0.73**
*Eam_2164*	*amsK*	amylovoran biosynthesis glycosyltransferase	2.21	0.81
*Eam_2163*	*amsL*	amylovoran biosynthesis protein	1.79	**1.05**
*Eam_2161*	*galE*	UDP-glucose 4-epimerase	1.48	0.57
*Eam_2162*	*galF*	UTP-glucose-1-phosphate uridylyltransferase	2.06	0.69
*Eam_1482*	*rcsA*	amylovoran biosynthesis regulator	**1.23**	0.28
*Eam_3468*	*lsc*	levansucrase	0.3	0.54
*Eam_2870*	*rlsA*	LysR-family transcriptional regulator	0.37	**1.18**
*Eam_3467*	*rlsB*	Levan regulatory protein	0.45	**1.27**
*Eam_0531*	*rlsC*	levan regulatory protein	0.47	1.39

Numbers in bold: p-value>0.05; all others with p-value<0.05. Expression ratio ≥2.0 indicates genes are up-regulated in mutants or overexpression strain and ≤0.5 indicates genes are down-regulated in mutants or overexpression strain.

Microarray data analyses revealed that when grown in MBMA medium, a total of 435 and 113 genes were significantly differentially expressed in the *amyR* mutant and in Ea1189 (pAmyR2) strain, respectively (Figure S1 and Table S1). Further analysis revealed that 82 genes were commonly differentially expressed in both the *amyR* mutant and Ea1189 (pAmyR2) in MBMA. Among these, 63 and 12 were either down- or up-regulated in both the *amyR* mutant and Ea1189 (pAmyR2) strain; whereas seven genes were inversely expressed in the *amyR* mutant and Ea1189 (pAmyR2) (Table S1).

Consistent with our previous observations [8], genes involved in amylovoran production (*amsABCDEGHIJK*) and regulation (*rcsA*) were significantly up-regulated in the *amyR* mutant in MBMA medium (Table 1). Amylovoran biosynthesis (*amsACFG*) and regulatory (*rcsA*) genes were slightly down-regulated in Ea1189 (pAmyR2) strain in MBMA medium (Table 1). Microarray results were consistent with qRT-PCR data as shown above (Fig. 1D). Expression of the *lsc* gene was down-regulated in both the *amyR* mutant and Ea1189 (pAmyR2) strain in MBMA (Table 1). However, three regulators of the *lsc* gene (*rlsABC*) were down-regulated only in MBMA in *amyR* mutant, but not in Ea1189 (pAmyR2) strain.

Furthermore, several groups of genes were analyzed among the differentially regulated genes (Table 2). Group I contained genes that had similar expression patterns as those for amylovoran biosynthesis and regulatory genes (Table 1). These included five glycogen biosynthetic genes (*glgABCPX*; *Eam_3268-3272*), one cell-wall biosynthetic gene (*wabP, Eam_1941*), and one membrane protein encoding gene (*Eam_0255*). Group II included genes from two operons; one encoding membrane proteins (*Eam_0322-0324*), while the other involved in histidine metabolism (*hutHGIU*; *Eam_1253-1259*). Genes in both operons were significantly up-regulated in both the *amyR* mutant and over-expression strains, as compared to Ea1189 in MBMA medium. The third group contains a single gene (*Eam_1299*), located immediately upstream of the *amyR* gene, encoding an oxygen-insensitive NADPH nitroreductase and involved in reduction of nitroaromatic compounds. This gene was significantly up-regulated in over-expression strains, but slightly down-regulated in the *amyR* mutant in MBMA (Table S1 and Table 2). In addition, many flagellar genes, including *flgBCDEFGMKL* and *fliCKLT* were down-regulated in both the *amyR* mutant and over-expression strains as compared to Ea1189 in MBMA medium; whereas, *flhDC* and *fliADSZ* were only down-regulated in the *amyR* mutant in MBMA (Table S1). Consistent with these data, motilities of both the *amyR* mutant and over-expression strains were decreased (Figure S2).

**Table 2:** Expression ratios of selected membrane, glycogen, and metabolism genes.

		Comparison	*ΔamyR*/WT	WT(pAmyR2)/WT
Gene	Name	Protein Description	MBMA	
**Group I** **Glycogen genes**				
*Eam_3268*	*glgP*	glycogen phosphorylase	4.09	0.67
*Eam_3269*	*glgA*	glycogen synthase	4.57	0.67
*Eam_3270*	*glgC*	glucose-1-phosphate adenylyltransferase	6.72	**0.66**
*Eam_3271*	*glgX*	glycogen debranching enzyme	4.67	0.62
*Eam_3272*	*glgB*	1,4-alpha-glucan branching enzyme	6.62	**0.7**
**Membrane protein/cell wall biosynthesis**				
*Eam_1941*	*wbaP*	UDP-Gal::undecaprenol phosphate Gal-1-P transferase	9.82	0.55
*Eam_0255*		putative membrane protein	3.75	0.41
**Group II**				
*Eam_0322*		Membrane protein	39.9	15
*Eam_0323*		Membrane protein	13.9	6.02
*Eam_0324*		Exported protein	7.31	3.1
**Histidine metabolism**				
*Eam_1253*	*hutU*	urocanate hydratase	3.18	4.47
*Eam_1254*	*hutH*	histidine ammonia-lyase	5.0	8.25
*Eam_1257*	*hutF*	putative chlorohydrolase	7.53	7.88
*Eam_1258*	*hutI*	imidazolonepropionase	11.58	9.27
*Eam_1259*	*hutG*	N-formylglutamate amidohydrolase	9.94	8.43
**Group III**				
*Eam_1299*	*nsfA*	Oxygen-insensitive NADPH nitroreductase	0.56	18.5

Numbers in bold: p-value>0.05; all others with p-value<0.05. Expression ratio ≥2.0 indicates genes are up-regulated in mutants or overexpression strain and ≤0.5 indicates genes are down-regulated in mutants or overexpression strain.

## Discussion

Thus far, genomes of three *E. amylovora* strains have been sequenced and published [4], [23]. Though *E. amylovora* has the smallest genome (about 3.8 Mbp) of enterobacteria sequenced until now (up to 5.5 Mbp), understanding the function of unknown genes, accounting for about half of annotated genes of any given genome, remains challenging. In this study, we have phenotypically characterized the roles of one conserved orphan gene, *amyR,* in *E. amylovora*, as well as determined the regulon of AmyR *in vitro*. AmyR, a homolog of *E. coli* YbjN, is an enterobacteria-specific orphan protein, which is evolutionally and functionally conserved. Cross-complementation experiments could restore phenotypes of both *E. coli ybjN* and *E. amylovora amyR* mutants (20). Results have shown that *amyR* in *E. amylovora* and *ybjN* in *E. coli* play similar roles in regulating exopolysaccharide production; however, there are marked differences in their roles in bacterial virulence and survival. This is possibly attributed to genotypic differences during evolution as well as adaptation to different environments and to different hosts.

In our previous studies, we have shown that *E. coli* YbjN and *E. amylovora* AmyR suppress colanic acid production in *E. coli* and amylovoran production in *E. amylovora*, respectively, suggesting they have conserved roles in controlling bacterial EPS production [8], [20]. Over-expression of *ybjN* or *amyR* in the WT strain or in many EPS over-producing mutant strains, such as *rcsC*, *ackA*, *envZ*, *ompR*, *grrS*, *grrA* and *hns* in either *E. coli* or *E. amylovora* also negatively influences EPS production [20], [24] (Fig. 2), thus suggesting that the role of YbjN/AmyR in regulating EPS production may reside downstream of these regulatory systems, and may also be independent of these systems, but with an unknown mechanism.

Regulation of EPS production in both *E. coli* and *E. amylovora* has been under intensive investigation [25], [26]. In our previous studies, we have identified several TCST systems involved in regulating amylovoran production [8], [12], [15]. Among these, the Rcs phosphorelay system, one of the most complicated and widely studied TCST systems, is essential for *E. amylovora* virulence [12]. The core of the Rcs system consists of the response regulator RcsB and the membrane-localized hybrid sensor kinase RcsC, which, upon sensing proper environmental stimulus/stimuli, autophosphorylates a conserved histidine residue in its histidine kinase domain. The *rcsC* mutant produces high levels of amylovoran *in vitro*, similar to that of the *amyR* mutant. Microarray analysis has identified more than 400 differentially expressed genes in both *rcsC* and *amyR* mutant compared to the WT strain *in vitro* [15, this study]. Among these, expression of amylovoran biosynthesis and related membrane protein-encoding genes, including *amsABCDEFGHJKL*, *glgABCPX*, *wbaP* (*Eam_1941*), *Eam_0255*, and *Eam0322_0324* (Tables 2 and 3), showed highly similar patterns in these two mutants, thus suggesting similar regulatory mechanisms may be shared by the two genes *in vitro*. However, the *rcsC* is non-pathogenic [12]; whereas, the *amyR* mutant is slightly more virulent than that of the WT strain (Fig. 4), suggesting that the regulatory mechanisms for RcsC and AmyR may be different *in vivo*.

**Table 3:** Bacterial strains, plasmids, and primers used in this study.

Strains, plasmids or primers	Relevant characters or sequences (5′—3′)^a^	Reference or source
Strains		
Ea1189	Wild type, isolated from apple	[32]
Ea273	Wild type, isolated from apple	[33]
Z2074*ΔamyR (ybjN)*	*amyR::Km*; Km^R^-insertional mutant of *amyR (ybjN)* of Ea1189, Km^R^	[8]
Z3207*ΔrcsC*	*rcsC::Km*; Km^R^-insertional mutant of *rcsC* of Ea1189, Km^R^	[8]
Z3742*ΔgrrS*	*grrS::Km*; Km^R^-insertional mutant of *grrS* of Ea1189, Km^R^	[8]
Z2198*ΔgrrA*	*grrA::Km*; Km^R^-insertional mutant of *grrA* of Ea1189, Km^R^	[8]
Z0270*ΔenvZ*	*envZ::Km*; Km^R^-insertional mutant of *envZ* of Ea1189, Km^R^	[8]
Z0271*ΔompR*	*ompR::Km*; Km^R^-insertional mutant of *ompR* of Ea1189, Km^R^	[8]
Z0118*Δhns*	*hns::Km*; Km^R^-insertional mutant of *hns* gene of Ea1189, Km^R^	[8]
*E. coli*		
DH10B	F^−^ *mcr*A Δ(*mrr*-*hsd*RMS-*mcr*BC) Φ80*lac*ZΔM15 Δ*lac*X74 *rec*A1 *end*A1 *ara*Δ139 Δ(*ara*, *leu*)7697 *gal*U *gal*K λ - *rps*L (Str^R^) *nup*G	Invitrogen
Plasmids		
pGEM ® T-easy	Ap^R^, PCR cloning vector, high copy number	Promega
pWSK29	Ap^R^; cloning vector, low copy number	[34]
pYbjN2(AmyR2)	1.1-kb PCR fragment containing *Erwinia amyR* gene in pGEM T-easy vector	[20]
pAmyR3	1.1 -kb EcoRI-BamHI fragment containing *Erwinia amyR* gene in pWSK29	This study
Primers^b^		
amyR3	CCGGAATTCGTTAGTGCATGAAAACTGTTACCG (EcoRI)	
amyR4	CGCGGATCCATAGCCCCAGTCATTCATGC (BamHI)	
amyREa1	TAATGGACGGGGTTATCCTG	
amyREa2	ATCAGCTTGGGCAGATTGTC	
16S3	CCTCCAAGTCGACATCGTTT	
16S4	TGTAGCGGTGAAATGCGTAG	
amsG1	CAAAGAGGTGCTGGAAGAGG	
amsG2	GTTCCATAGTTGCGGCAGTT	
amsC1	CTGGCATGGATGATTCACAG	
amsC2	CTCTCATGGGTGAAACACGA	
amsD1	GATGCGTCTGTTCAAGCTGT	
amsD2	TCGCAACAAATCAGTCTGGA	
rcsA1	TTAAACCTGTCTGTGCGTCA	
rcsA2	AGAAACCGTTTTGGCTTTGA	

a Km^R^, Ap^R^ and Str^R^ = kanamycin, ampicillin and streptomycin resistance, respectively.

b Underlined nucleotides are restriction sites added and the restriction enzymes are indicated at the end of primers.

In addition to affecting EPS production, mutation of *ybjN* in *E. coli* resulted in increased motility, fimbriation (auto-aggregation), and biofilm formation; whereas, over-expression of *ybjN* in *E. coli* led to reduced motility, fimbriation, biofilm formation, and resistance to acidic conditions [20], [27]. However, we did not observe similar phenotypic changes in *E. amylovora* either in the *amyR* mutant or in the *amyR* over-expression strain, except for slight reduced motility of both the *amyR* mutant and over-expression strains (Figure S2). Moreover, microarray analysis confirmed that many flagellar genes were down-regulated in both the *amyR* mutant and over-expression strains when compared to WT (Table S1); whereas, flagellar genes in *E. coli* were up-regulated in the *ybjN* mutant, but down-regulated in over-expression strains *in vitro* [20]. In addition, an operon (*hutHGIU*; *Eam_1253-1259*) involved in histidine metabolism was highly expressed in both the *amyR* mutant and over-expressing strains as compared to WT (Table 2). The first three steps for degradation of histidine involve activities of HutH, HutU, and HutI (*Eam_1253-1254, Eam_1258*) by converting L-histidine to N-formimino-L-glutamate. Hydrolase encoded by *Eam_1257* (HutF) and formylglutmase encoded by HutG (*Eam_1259*) then convert formiminoglutamate into glutamate and formate [28]. However, questions as to why this operon is strongly induced and what is the role of histidine metabolism in *E. amylovora* are yet to be answered.

Conversely, over-expression of *ybjN* in *E. coli* suppresses bacterial growth in liquid medium [20]. However, in *E. amylovora*, though over-expression of *amyR* did not affect bacterial growth in medium, both mutation of *amyR* and over-expression of *amyR* in *E. amylovora* have affected bacterial virulence *in vivo* and bacterial growth on immature pear fruit. To our surprise, over-expression of *amyR* in Ea1189 and Ea273 resulted in non-pathogenic on apple shoots and leaves. Moreover, over-expression of *ybjN* in *E. coli* leads to identifying large numbers of differentially expressed genes, including down-regulation of metabolic pathways and up-regulation of stress-related genes [20]; whereas in *E. amylovora*, mutation of *amyR*, rather than over-expression of *amyR*, has led to identifying large numbers of differentially modulated genes *in vitro*. These discrepancies may suggest that diversification of the *amyR/ybjN* gene function exits in enterobacteria, possibly due to evolution and adaptation to different environments and hosts.

In summary, the findings reported in this study present the first global view of the likely functions of an unknown orphan gene, *amyR*, in one of the most important plant pathogenic bacteria, which may provide direction for future studies to determine the molecular mechanism and biochemical function of the orphan protein AmyR/YbjN. Our results have clearly demonstrated that the YbjN family in enterobacteria has evolved in regulating bacterial EPS production, and has also diversified their roles in bacterial survival, metabolism, and virulence in different bacterial species.

## Materials and Methods

### Bacterial stains and culture media

Bacterial strains and plasmids used in this study are listed in Table 3. The LB medium is used routinely for culturing *E. amylovora*. When necessary, the following antibiotics were added to the medium: 50 µg ml^−1^ kanamycin and 100 µg ml^−1^ ampicillin. Amylovoran production was determined by growing bacteria in MBMA medium(3 g KH_2_PO_4_, 7 g K2HPO_4_, 1 g [NH_4_]_2_SO_4_, 2 ml glycerol, 0.5 g citric acid, 0.03 g MgSO_4_) plus 1% sorbitol [29].

### DNA manipulation and sequence analysis

Plasmid DNA purification, PCR amplification of genes, isolation of fragments from agarose gels, cloning, and restriction enzyme digestion and T4 DNA ligation were performed using standard molecular procedures [30].

### Cloning *amyR* gene from *E. amylovora*

The *amyR* gene was cloned into a low-copy number vector, pWSK29. Primer pair amyR3-amyR4 containing *EcoR*I and *BamH*I restriction sites were used to amplify the *amyR* gene and its flanking sequences. Following amplification, DNA fragments and the vector were both digested by *EcoR*I and *BamH*I and ligated together. The final plasmid was designated as pAmyR3. All plasmids were introduced into *E. amylovora* strains by electroporation. Transformants were selected on LB plates supplemented with Km and Ap. Their genotypes were confirmed by both enzymatic digestion and sequencing.

### Virulence assays on apple plants

Virulence tests on shoots of apple cv. Gala were performed as described previously [21]. Briefly, young shoots of 22–25 cm in length, were pricked with a needle at the tip and 5 µl of bacterial suspension (OD_600_ = 0.1) was inoculated on the wound tissue. For each bacterial strain, 8–10 shoots were inoculated. Plants were kept in a greenhouse at 25°C and 16 h light photoperiod. Disease severity was measured as the average length of necrotic symptoms on apple shoots for up to 8 days. The experiment was repeated two times with similar results.

Virulence assays were also carried out on leaves of apple cv. Red Delicious as described previously [21]. Briefly, 9 to 10 young leaves, 3 to 4 cm in length, were inoculated with scissors dipped in bacterial suspension (OD_600_ = 0.1). Plants were kept in a greenhouse at 25°C and 16 h light photoperiod. The disease severity was evaluated and recorded for up to eight dpi. Progression of necrosis was recorded using an arbitrary scale as described previously [21] as follows: 0, no necrosis; 1, necrosis limited to the inoculation point; 2, necrosis reaching the midrib; 3, necrosis reaching the petiole; 4, necrosis reaching the stem; 5, necrosis on the main shoot. For each inoculated strain, the necrosis severity index (NSI) was calculated using the following formula: 
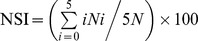
, wherein *N_i_* corresponded to number of leaves with disease severity of *i*; and *N* was the sample size. The experiment was repeated two times with similar results.

### Immature pear virulence assays

For different *E. amylovora* strains, bacterial suspensions were grown overnight in LB broth, harvested by centrifugation, and resuspended in 0.5× sterile phosphate buffered-saline (PBS) with bacterial cells adjusted to concentrations of ∼1×10^3^ to 1×10^4^ colony-forming units (cfu/µl) (OD_600_ = 0.1 and then diluted 100-fold) in PBS. Immature pear fruits (*Pyrus communis* L. cv. ‘Bartlett’) were surface-sterilized and pricked with a sterile needle as described previously [6], [7]. Three wounded fruits were inoculated with 2 µl of cell suspensions for each strain, and incubated in a humid chamber at 26°C. Symptoms were recorded at 4, 6 and 8 dpi.

For bacterial population studies, fruit tissues surrounding the inoculation site were excised using a #4 cork borer as described previously [6], [7], and homogenized in 0.5 ml of 0.5× PBS. Bacterial growth was monitored by dilution-plating on LB medium amended with the appropriate antibiotics. For each strain tested, fruits were assayed in triplicate, and each experiment was repeated at least twice.

### CPC assay for determining amylovoran concentration

The amylovoran concentration in supernatants of bacterial cultures was quantitatively determined by a turbidity assay with cetylpyrimidinium chloride (CPC) as previously described [8], [29]. Briefly, bacterial cells were grown overnight in LB broth w/o appropriate antibiotics, harvested by centrifugation, and washed with PBS three times. After the final wash, the pellet was resuspended in 200 µl PBS. A total of 100 µl of bacterial suspension was inoculated into 10 ml MBMA medium with 1% sorbitol. One ml of bacterial cells was pelleted two days after inoculation at 28°C with shaking. Following centrifugation, 50 µl CPC at 50 mg ml^−1^ was added to 1 ml supernatant. After 10 min of incubation at room temperature, the amylovoran concentration was determined by measuring OD_600_ turbidity. The final concentration of amylovoran production was normalized for a cell density of 1.0. For each strain tested, the experiment was repeated at least three times.

### Motility assay

For *E. amylovora* WT, mutant strain and over-expression strains, bacterial suspensions were grown overnight in LB broth with appropriate antibiotics, harvested by centrifugation, washed with PBS once, and resuspended in 200 µl PBS. Then, bacterial suspensions were diluted 10× in water, and 5 µl of the diluted bacterial suspension was plated onto the center of agar plates (10 g tryptone, 5 g NaCl, 3 g agar per l Liter) as previously described [8]. Diameters were determined following incubation at 28°C for up to three days. The experiments were repeated at least three times.

### Levasucrase activity assay

To determine levasucrase activity, bacterial cells were grown in LB medium at 28°C for 24 h. Supernatants were mixed with equal volume of LS-Buffer as described previously [31] and incubated at 28°C for 24 h. Turbidity was measured at 600 nm and normalized for a cell density of 1. The experiments were performed at least 3 times giving similar results.

### RNA isolation

Bacterial strains were grown overnight in LB medium and diluted in 5 ml MBMA medium at an OD_600_ of 0.005. After 18 h growth in MBMA medium at 28°C, 2 ml of RNA Protect Reagent (Qiagen) was added to 1 ml bacterial culture (at OD_600_ of about 0.5–0.8) to stabilize RNA. Cells were harvested by centrifugation for 10 min at 4000 *g* and RNA was extracted using Qiagen Bacterial RNA Protect Mini Kit as recommended by the manufacturer (Qiagen, Hilden, Germany). On-column DNA digestion was performed using Qiagen DNase. RNA was quantified using Nano-Drop ND-100 spectrophotometer (NanoDrop Technologies; Wilminton, DE) and RNA quality was checked using the Agilent 2100 Bioanalyzer (Agilent Technologies, Palo Alto, CA, USA).

### Microarray hybridization and data analysis

Transcriptome profiling was performed as described previously [15], [20]. A 60-mer *E. amylovora* microarray (8×15 K) based on Ea273 genome sequence was purchased from Agilent, and as described previously [5], [15]. Each slide contains 8 arrays and each array has nearly 15,000 spots, containing each probe in triplicate. The microarray design is available at ArrayExpress (http://www.ebi.ac.uk/arrayexpress/, accessions: Microarray #A-MEXP-2000). All microarray data are available at NCBI Gene Expression Omnibus (GEO) (http://www.ncbi.nlm.nih.gov/geo, accession #GSE37064). Three biological replicates for each mutant and overexpression strain and two biological replicates for WT strain were hybridized to two or three arrays and cross compared as technical replicates.

Ten µg of total RNA from each sample were reverse transcribed and labeled by Alexa Fluor dye 555 using the FairPlay III Microarray Labeling Kit (Stratagene, La Jolla, CA, USA) according to manufacturer's instruction, except that purification steps were done using the QIAquick PCR Purification Kit (Qiagen, Hilden, Germany). Microarray hybridization was performed using 600 ng dye-labeled cDNA in the presence of a 2× Hybridization Buffer (Agilent Technologies, Palo Alto, CA, USA) for 17 h at 65°C in an Agilent rotating oven (10 rpm). Microarray slides were then washed for 1 min in Gene Expression Wash Buffer 1 at room temperature, and another 1 min in Gene Expression Wash Buffer 2 (Agilent technologies, Palo Alto, CA, USA) at 37°C. Slides were scanned using an Axon 4000B Array Scanner at 5-µm resolution. PMT voltages were automatically adjusted using the Genepix Pro 6.0 software acquisition system to obtain maximal signal intensities with <0.02% probe saturation. The resulting 16 bit images were processed using the GenePix Pro 6.0 image analysis software (v6.0.1.26). Raw data were logarithmically transformed and normalized using the glowess method by R software (R.2.2.1).

Statistical comparisons were performed using multiple testing procedures to evaluate statistical significance for differentially expressed genes. A modified t-test was computed to measure the significance associated with each differential expression value. A gene expression value was decided to be significantly different when the p-value was less than 0.05 (except otherwise mentioned) and the expression ratio was ≥2.0 or ≤0.5. Gene functions were assigned using data from NCBI (http://www.ncbi.nlm.nih.gov).

### Quantitative real-time PCR (qRT-PCR)

To validate microarray data, qRT-PCR was performed for selected target genes. Approximately 1 µg of total RNA was reverse-transcribed in a 20 µl reaction using SuperScript® III Reverse Transcriptase (Invitrogen, Carlsbad, CA, USA) following the manufacturer's instructions. For each sample, negative reverse transcription reaction was done to verify the absence of genomic DNA contamination in subsequent qRT-PCR. Primers used for quantitative real time PCR (qRT-PCR) in this study are listed in Table 3. Primer sequences were designed using Primer3 (http://frodo.wi.mit.edu/primer3/). BLAST searches were performed to confirm gene specificity and the absence of multi-locus matching at the primer site. SYBRGreen reactions were performed using the ABI 7300 System (Applied Biosystems, Foster City, CA) in 96 well optical reaction plates. cDNA was precipitated in ice-cold 100% ethanol at −20°C, resuspended in water, and quantified using Nano-Drop ND-100 spectrophotometer (NanoDrop Technologies; Wilminton, DE). 1 µl of cDNA (2 ng/reaction) or water (no-template control) were used as template for qRT-PCR reactions with SYBR Fast Green PCR Master Mix (Applied Biosystems, Foster City, CA) and primers at 500 nM final concentration were added. Primer pairs amyREa1-amyREa2, 16S3–16S4, amsG1-amsG2, amsC1-amsC2, amsD1-amsD2, and rcsA1-rcsA2 were used to detect expression of *amyR*, *rrsA*, *amsG*, *amsC*, *amsD*, and *rcsA* genes, respectively. qRT-PCR amplifications were carried out at 50°C for 2 min, 95°C for 10 min, followed by 40 cycles of 95°C for 15 sec and 60°C for 1 min, and a final dissociation curve analysis step from 65°C to 95°C. Technical replicate experiments were performed for each biological triplicate sample. Amplification specificity for each reaction was confirmed by the dissociation curve analysis. Determined Ct values were then exploited for further analysis.

Gene expression levels were analyzed using the relative quantification (ΔΔ-Ct method) [15]. A 16S rDNA (rrsA) gene was used as an endogenous control. A relative quantification (RQ) value was calculated for each gene with the control group as a reference. A p-value was computed using a moderated t-test to measure the significance associated with each RQ value. Variations were considered statistically significant when the p-value was <0.05. RQ values were then normalized to those of WT or control medium.

## Supporting Information

Figure S1 **Venn Diagram showing number of differentially regulated genes in MBMA medium for** ***amyR*** **/WT and WT(pAmyR2)/WT.**
(EPS)

Figure S2 **Bacterial motility on 0.3% agar plates.** A. Effect of *amyR* mutation or over-expression on motility. Cells were spotted at the center of the plate (0.3% agar) and incubated at 28°C for 48 h. B. Comparison of the movement distance of different strains. For irregular movement, we measured the greatest diameter. 1: Ea1189, 2: *ΔamyR*, 3: *ΔamyR* (pAmyR2), 4: Ea1189 (pAmyR3), 5: Ea1189 (pAmyR2), 6: Ea273, 7: Ea273 (pAmyR3), 8: Ea273 (pAmyR2). Data points represent means of three replicates ± standard deviations. Similar results were obtained in three independent experiments.
(EPS)

Table S1 **Differentially expressed genes in** ***amyR*** **mutant and Ea1189 (pAmyR2) compared to Ea1189 in MBMA medium.**
(XLSX)
